# Tropical Peat and Peatland Development in the Floodplains of the Greater Pamba Basin, South-Western India during the Holocene

**DOI:** 10.1371/journal.pone.0154297

**Published:** 2016-05-10

**Authors:** Navnith K. P. Kumaran, Damodaran Padmalal, Ruta B. Limaye, Vishnu Mohan S., Tim Jennerjahn, Pradeep G. Gamre

**Affiliations:** 1 Biodiversity and Palaeobiology Group, Palynology and Palaeoclimate Laboratory, Agharkar Research Institute, Pune—411004, India; 2 National Centre for Earth Science Studies, Thuruvaikkal P.O., Thiruvananthapuram—695011, Kerala, India; 3 Leibniz Centre for Tropical Marine Ecology (ZMT), Biogeochemistry and Geology, Bremen—28359, Germany; Institute of Botany, CHINA

## Abstract

Holocene sequences in the humid tropical region of Kerala, South-western (SW) India have preserved abundance of organic—rich sediments in the form of peat and its rapid development in a narrow time frame towards Middle Holocene has been found to be significant. The sub—coastal areas and flood plains of the Greater Pamba Basin have provided palaeorecords of peat indicating that the deposits are essentially formed within freshwater. The combination of factors like stabilized sea level and its subsequent fall since the Middle Holocene, topographic relief and climatic conditions led to rapid peat accumulation across the coastal lowlands. The high rainfall and massive floods coupled with a rising sea level must have inundated > 75% of the coastal plain land converting it into a veritable lagoon—lake system that eventually led to abrupt termination of the forest ecosystem and also converted the floodplains into peatland where accumulation of peat almost to 2.0–3.0 m thickness in coastal lowlands and river basins during the shorter interval in the Middle Holocene. Vast areas of the coastal plains of Kerala have been converted into carbon rich peatland during the Middle Holocene and transforming the entire coastal stretch and associated landforms as one of the relatively youngest peatlands in the extreme southern tip of India. Unlike the uninterrupted formation of peatlands of considerable extent during the Holocene in Southeast Asia, the south Peninsular Indian region has restricted and short intervals of peatlands in the floodplains and coastal lowlands. Such a scenario is attributed to the topographic relief of the terrain and the prevailing hydrological regimes and environmental conditions as a consequence of monsoon variability since Middle Holocene in SW India. Considering the tropical coastal lowlands and associated peatlands are excellent repositories of carbon, they are very important for regional carbon cycling and habitat diversity. The alarming rate of land modification and development is destabilizing these carbon pools resulting in large scale carbon emissions to the atmosphere and loss of low-latitude peat palaeorecords. Therefore, these palaeorecords are to be conserved and addressed for better understanding and utilizing the carbon pool for effective climate change adaptation. This communication is the first attempt of addressing the peat formation and peatland development during the Holocene from the tropical region of Peninsular India.

## 1. Introduction

Peat deposits are excellent natural archives that have been widely used for palaeoclimate reconstruction in tropical and temperate regions. Peat is formed in wetlands where flooding obstructs flow of oxygen from the atmosphere and creates anoxic conditions which allow low rates of decomposition of organic detritus. Slow accumulation of partially decayed vegetation in such an environmental set up leads to formation of unique natural areas called peatlands. The peatland ecosystem plays a pivotal role in carbon cycle because peatland plants capture atmospheric CO_2_ in substantial quantities which is naturally released back to the atmosphere during early diagenesis of peat, thus maintaining the natural equilibrium. Since organic matter accumulation in peatlands continues for thousands of years, peat deposits offer excellent records of past vegetation and climates stored in plant remains, especially the pollen grains. Although a wealth of information exists on global peats, tropical peat deposits in Indian subcontinent received very little attention except peat bog of Late Pleistocene from the Nilgiri Hills, a mountain region more than 2,000 m a.s.l. in southern India [1] and that of Himalayan Basin [2]. Despite its occurrence in the Late Quaternary sequences in most of the sedimentary basins of India palaeoclimate potential of this unique sedimentary archive has been seldom explored. Considering the occurrence of peat within the time frame of Early to Middle Holocene at many locations (8. 0–5.0 k yrs BP) and all along the coastline and especially in the lowlands during the Middle Holocene (6.0–5.0 k yrs BP) an attempt is made to focus on the peatland development in this part of the tropical land.

Peat has been found to be a key bed in the Late Quaternary sedimentary deposit of Kerala (India) and it is essentially derived from the tropical evergreen forests and fresh water swamps of lowland and midland during early Middle Holocene [3]. However, it has also been reported from the coastal wetlands associated with the lagoons, paleo—delta and creeks [4–6] and in the form of reworked Neogene deposits [3]. Despite its wide spread occurrence and imparting black colour to the Late Quaternary and recent sediments, significance of peat and development of tropical peatland in Peninsular India in general and in SW India in particular has not been dealt. Accordingly, the retrieved data of peat sequences obtained from the subsurface sediments and peatland development since Middle Holocene in the floodplains of river basins in Pamba region have been addressed in the present communication. The relevance of peat and importance of tropical peatland in issues related to palaeoclimate study based on subsurface data using multiple proxies from Achankovil and Pamba river basins (Greater Pamba Basin) in Kerala have been presented here.

## 2. Geological and environmental settings Pamba–Achankovil—Manimala River systems (Greater Pamba Basin)

**Fig 1:**
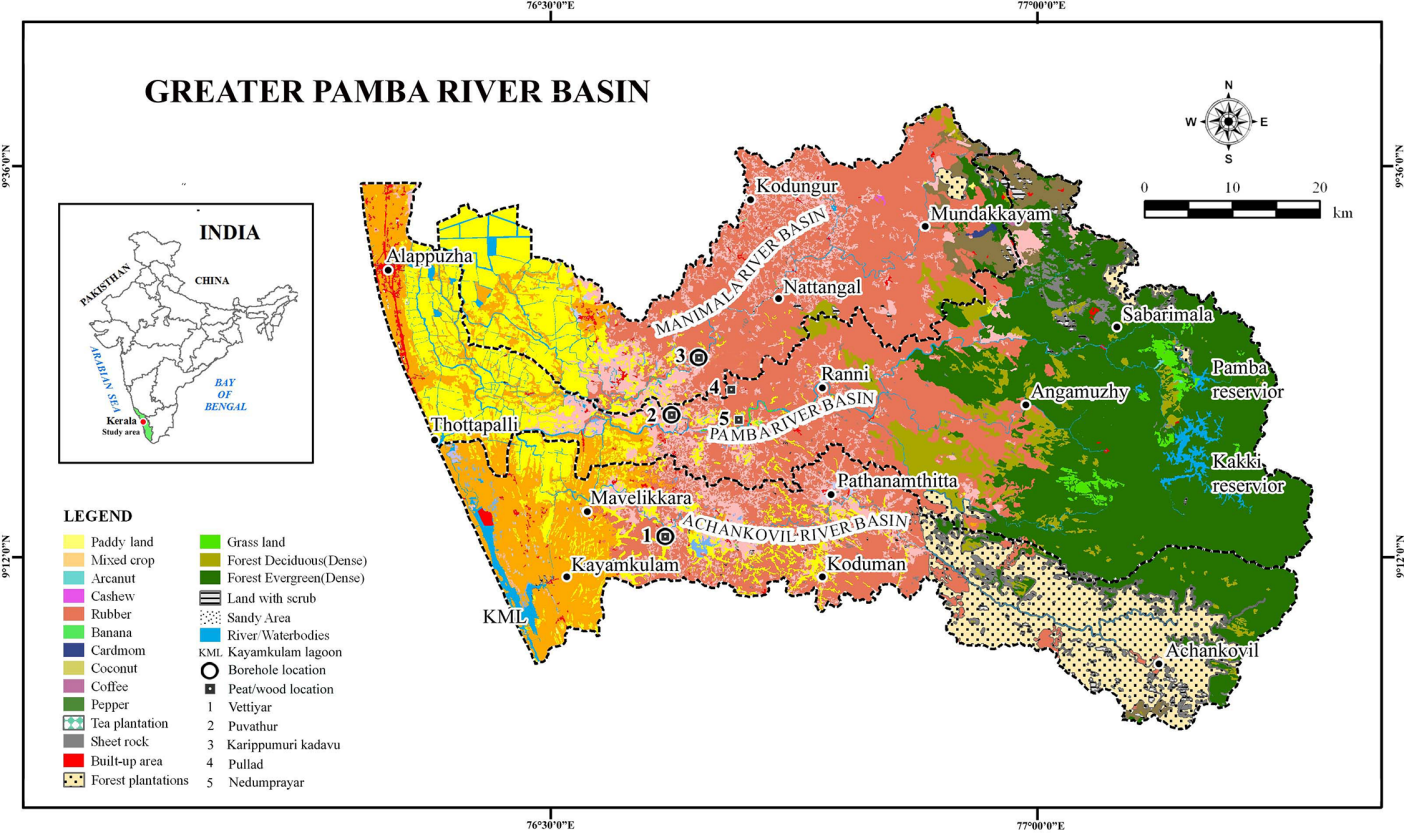
Land use / Land cover map of Greater Pamba River Basin (Achankovil, Pamba and Manimala rivers) showing locations of peat/wood deposits and borehole cores.

The Greater Pamba Basin (GPB) refers to the entire area drained by the Pamba, Manimala and Achankovil rivers is very important in socio—economic and cultural perspective of Kerala State in south-western India. Of the 44 rivers, Pamba had been the longest river in the erstwhile princely state of Travancore, but now is the third longest river in the state. Pamba River originates at Pulachimalai hill in the Peerumedu plateau (Idukki District) in the Western Ghats at an altitude of 1,650 m a.s.l. and flows through several places in Pathanamthitta and Alappuzha districts including Kuttanad, an important rice cultivating centre, before emptying into the Vembanad Lake. The basin extends over an area of 2,235 sq km with the entire catchment area limited to Kerala state and is bounded on the east by Western Ghats and on the west by Arabian Sea (Fig 1). The salient drainage characteristics of the Manimala and Achankovil rivers that form respectively the northern and southern boundaries are summarised in Table 1. In the highlands and eastern part of the midland, the rivers drain over a spectrum of rock types which include pyroxene granulites, charnockites and khondalites as the major constituents. Its course in the midland region (7.5 to 75 m above sea level) is mainly through laterites and Holocene sediments. In the lowland (< 7.5 m above sea level) the rivers flow through alluvial sands and clays of Holocene age. The rivers display dendritic and trellis drainage patterns in higher altitudes. On entering the coastal plains, the rivers take a northward trend and join the Vembanad Lake at Pallathuruthy near Alappuzha. The present northward trend in the lowlands has been caused by the silting of this water body coupled with a northward tilt during Late Pleistocene—Early Holocene [7–9].

**Table 1:** Salient features of the rivers draining Greater Pamba Basin.

Sr. No	Rivers	Drainage characteristics					
		River length (km)	Catchment area (km^2^)	Stream order	HWE (m)	Drainage density (km/km^2^)	River type *
1	Achankovil	128	1484	7	700	1.94	Upland
2	Pamba	176	2235	6	1650	0.30	Mountainous
3	Manimala	90	847	6	1156	0.29	Mountainous

HWE Head water elevation * [40].

Monsoon variability and anthropogenic factors in tandem have brought in substantial modification of the hydrological regimes leading to significant changes in landforms and forest cover in south-western India. Historical records and satellite imageries showed that the Pamba River had been vigorous and well linked for navigation, freshwater supply and cultural feats in the erstwhile princely state. However, the current status of Pamba River is alarming as it has shrunk considerably to a stream and is totally dry at many places. In view of the above alarming situation it is mandatory to decode the past signatures that are preserved in the sedimentary archives for addressing the natural and anthropogenic factors that led to the deterioration of Pamba River and its ecology. The subsurface sediment profile of about 10.50 m long core from Vettiyar (Achankovil River Basin), 12.50 m long core from Puvathur (Pamba River Basin), and 10.5 m long core from Karippumuri kadavu (Manimala River Basin) have provided adequate terrestrial archives in the form of peat deposits and freshwater swamp sub-fossil logs as proxies to decode climate variability and geological events that led to the changing scenario of the Greater Pamba Basin and its forest cover (Fig 1; Table 2).

**Table 2:** Sedimentological characteristics of Vettiyar, Puvathur and Karippumuri kadavu borehole cores.

BoreholeLocation	Depth (cm)	Sand (%)	Silt (%)	Clay (%)	C-org(%)	Sediment type ^(1)^	Remarks
**Vettiyar**	50–60	15.91	27.27	56.82	NA	zC	Greyish black, organic matter rich sediments with sands of fine and very fine grade
	90–100	19.2	26.24	54.56	NA	zC	Greyish black, organic matter rich sediments with sands of fine and very fine grade
	210–220	17.99	28.06	53.95	6.66	zC	Greyish black, organic matter rich sediments with sands of fine and very fine grade
	270–280	20.74	35.63	43.63	3.22	cM	Greyish black, organic matter rich sediments with sands of fine and very fine grade.
	390–300	1.89	24.4	73.71	5.79	zC	Greyish black, organic matter rich sediments with sands of fine and very fine grade.
	340–350	NA	NA	NA	11.75	Peat	Decayed vegetal remains with wood fragments.
	390–400	NA	NA	NA	23.9	Peat	Decayed vegetal remains with wood fragments.
	460–470	NA	NA	NA	5.89	Peat	Decayed vegetal remains with wood fragments.
	490–500	34.23	13.35	52.42	1.12	sC	Greyish black sediments with medium to fine grade sand; opaques and sillimanite dominate the heavy mineral residue; traces of zircon and rutile are also seen.
	540–550	35.96	7.35	56.69	0.29	sC	Greyish black sediments with medium to fine grade sand; opaques and sillimanite dominate the heavy mineral residue; traces of zircon and rutile are also seen.
	590–600	44.25	2.57	53.18	0.41	sC	Greyish black sediments with medium to fine grade sand; opaques and sillimanite dominate the heavy mineral residue; traces of zircon and rutile are also seen.
	640–650	46.78	9.75	43.47	0.3	sM	Greyish white sediments with medium to fine grade sand; opaques and sillimanite dominates the heavy mineral residue; traces of zircon are also seen.
	690–700	47.38	3.79	48.83	0.26	cM	Greyish white sediments with medium to fine grade sand; opaques and sillimanite dominates the heavy mineral residue; traces of zircon are also seen.
	740–750	57.9	16.96	25.14	0.23	cS	Reddish brown sediments with sands of coarser grade; opaques and sillimanite dominate the heavy mineral residue; traces of zircon and monazite are also seen.
	790–800	56.05	0.52	43.43	0.03	cS	Reddish brown sediments with sands of coarser grade; opaques and sillimanite dominate the heavy mineral residue; traces of zircon and monazite are also seen.
	960–970	54.63	4.53	40.84	0.12	cS	Reddish brown sediments with sands of coarser grade; opaques and sillimanite dominate the heavy mineral residue; traces of zircon and monazite are also seen.
**Puvathur**	10–20	59.72	17.86	22.42	0.82	cS	Yellowish to greyish brown sediments with high content of fine sands; heavy mineral residue contains opaques and inosilicates as major minerals; traces of garnet, biotite and zircon are also seen.
	90–100	65.92	15.2	18.88	0.5	cS	Yellowish to greyish brown sediments with high content of fine sands; heavy mineral residue contains opaques and inosilicates as major minerals; traces of garnet, biotite and zircon are also seen.
	190–200	57.05	18.06	24.89	0.58	cS	Yellowish to greyish brown sediments with high content of fine sands; heavy mineral residue contains opaques and inosilicates as major minerals; traces of garnet, biotite and zircon are also seen.
	290–300	62.89	16.81	20.3	0.24	cS	Yellowish to greyish brown sediments with high content of fine sands; heavy mineral residue contains opaques and inosilicates as major minerals; traces of garnet, biotite and zircon are also seen.
	390–400	40.47	22.66	36.87	0.53	sM	Greyish brown to black sediments; sands are generally of coarser to medium grade; opaques and inosilicates dominate the heavy mineral residue; traces of garnet, sillimanite, biotite and zircon are also seen.
	490–500	87.94	3.27	8.79	0.13	S	Greyish brown to black sediments; sands are generally of coarser to medium grade; opaques and inosilicates dominate the heavy mineral residue; traces of garnet, sillimanite, biotite and zircon are also seen.
	590–600	5.56	46.29	48.15	1.97	cM	Greyish brown to black sediments; sands are generally of coarser to medium grade; opaques and inosilicates dominate the heavy mineral residue; traces of garnet, sillimanite, biotite and zircon are also seen.
	690–700	NA	NA	NA	26.15	Peat	Decayed vegetal remains with occasional presence of twigs and wood fragments.
	790–800	NA	NA	NA	11.56	Peat	Decayed vegetal remains with occasional presence of twigs and wood fragments.
	890–900	3.46	37.53	59.01	9.86	zC	Greyish black, organic matter rich sediments with decayed wood.
	900–1000	0.56	29.41	70.03	4.52	zC	Greyish black, organic matter rich sediments with decayed wood.
	1090–1100	16.35	27.33	56.32	2.83	zC	Greyish black sediments with sands of coarser grade; opaques and garnet dominate the heavy mineral residue; Zircon, sillimanite, inosilicates and biotite are seen as traces.
	1140–1150	50.14	17.48	32.38	2.87	cS	Greyish black sediments with sands of coarser grade; opaques and garnet dominate the heavy mineral residue; Zircon, sillimanite, inosilicates and biotite are seen as traces.
	1240–1250	Altered rock	Altered rock	Altered rock			Weathered basement rock with quartzose sands of coarser grade; opaques and garnet dominates the heavy mineral residue; Zircon, sillimanite, inosilicates and biotite are seen as traces.
**Karippumuri kadavu**	0–10	22.42	35.43	42.15	0.72	cM	Yellowish brown sandy mud with sands of fine to very fine grade.
	90–100	21.05	33.63	45.32	0.32	cM	Yellowish brown sandy mud with sands of fine to very fine grade
	140–150	38.4	41.15	20.45	0.69	zM	Yellowish brown sandy mud with sands of fine to very fine grade
	240–250	35.33	44.56	20.11	0.42	zM	Yellowish brown sandy mud with sands of fine to very fine grade
	340–350	36.34	43.21	20.45	0.72	zM	Yellowish brown sandy mud with sands of fine to very fine grade
	390–400	37.21	41.3	21.49	0.52	zM	Yellowish brown sandy mud with sands of fine to very fine grade
	440–450	90.94	2.16	6.9	0.35	S	Yellowish brown to light grey, medium to coarse sands; heavy mineral residue contains opaques and inosilicates as major minerals; traces of zircon, monazite, garnet and sillimanite are also seen.
	490–500	88.96	1.83	9.21	0.43	S	Yellowish brown to light grey, medium to coarse sands; heavy mineral residue contains opaques and inosilicates as major minerals; traces of zircon, monazite, garnet and sillimanite are also seen.
	540–550	85.22	5.57	10.29	0.33	S	Yellowish brown to light grey, medium to coarse sands; heavy mineral residue contains opaques and inosilicates as major minerals; traces of zircon, monazite, garnet and sillimanite are also seen.
	590–600	80.21	7.12	12.67	0.45	S	Yellowish brown to light grey, medium to coarse sands; heavy mineral residue contains opaques and inosilicates as major minerals; traces of zircon, monazite, garnet and sillimanite are also seen.
	640–650	22.12	33.52	44.36	1.12	cM	Greyish black, organic matter bearing sediments; sands are of fine to very fine grade.
	690–700	25.31	31.4	43.29	1.99	cM	Greyish black, organic matter bearing sediments; sands are of fine to very fine grade.
	740–750	19.21	32.42	48.37	2.19	cM	Greyish black, organic matter bearing sediments; sands are of fine to very fine grade.
	790–800	NA	NA	NA	10.1	Peat	Decayed vegetal remains with occasional presence of wood fragments.
	840–850	NA	NA	NA	18.99	Peat	Decayed vegetal remains with occasional presence of wood fragments.
	890–900	NA	NA	NA	12.01	Peat	Decayed vegetal remains with occasional presence of wood fragments.
	910–920	66.45	29.31	4.24	2.23	zS	Greyish black, organic matter rich sediments with sands of medium to coarse grade.
	990–1000	Altered rock	Altered rock	Altered rock			Weathered rock basement; opaques and garnet dominates the heavy mineral residue; Zircon and sillimanite are seen as traces.

^(1)^[15]; NA-Not Analysed; S—Sand; zS Silty sand; cS—Clayey sand; sM—Sandy mud; cM—Clayey mud; zM Silty mud; zC—Silty clay, sC—Sandy clay.

## 3. Material and Methods

**Fig 2:**
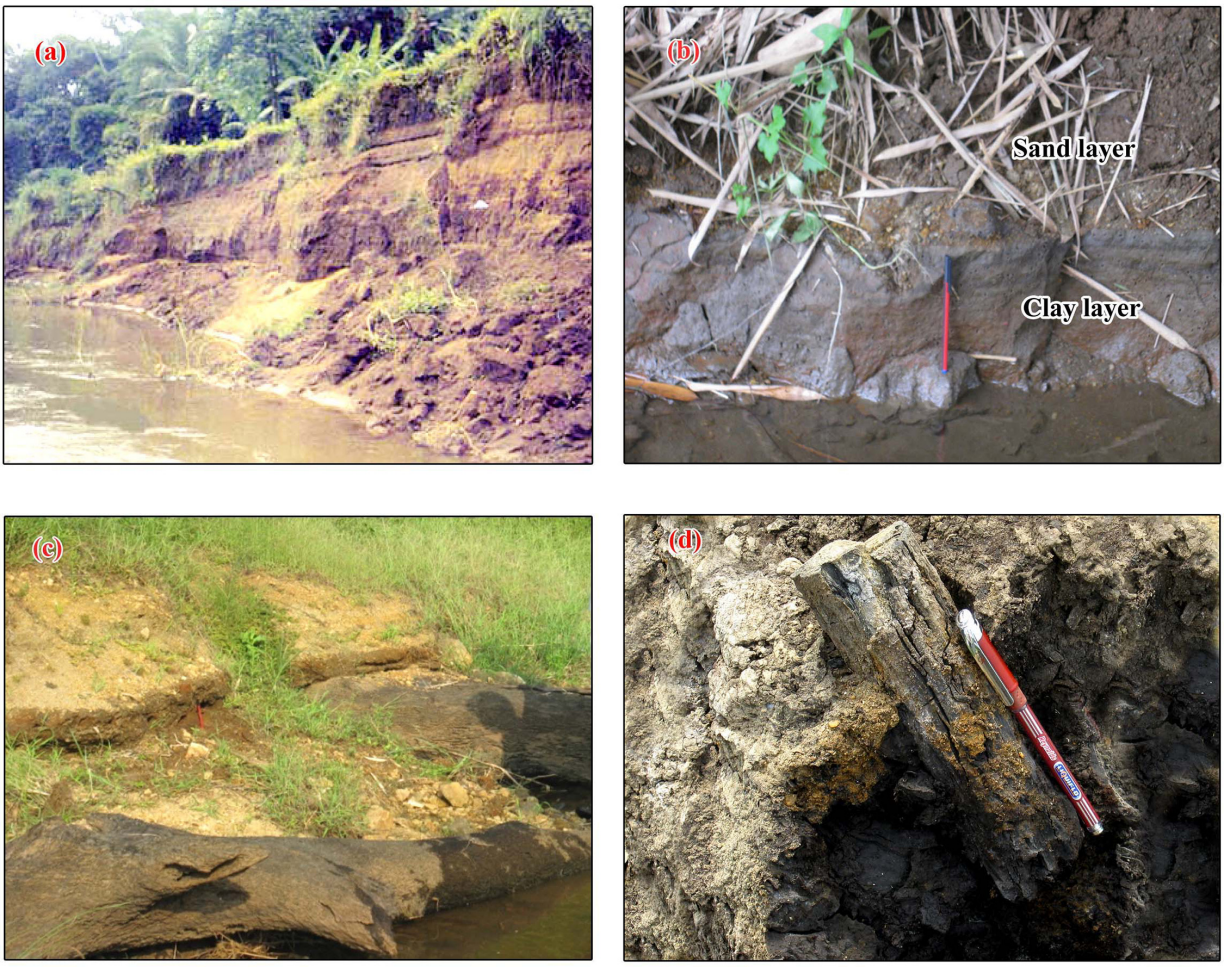
Selected field locations and sections from the study area—(a) Pamba river bank exposing yellowish brown, silty mud with intercalations of sand (b) Carbonaceous clay—sand sequence exposed on the bank of Manimala River near Karippumuri kadavu (c) Huge tree trunks exposed on bottom of the bank of Manimala River (d) A piece of wood embedded in the carbonaceous clays excavated from the Achankovil river basin (near Vettiyar) during the process of a canal construction through its floodplain.

The authors obtained necessary permit to carry out field work from the respective heads of the organizations, namely, Director, Agharkar Research Institute, Pune and National Centre for Earth Science Studies, Thiruvananthapuram. Accordingly, no specific permissions were required to carry out field work and drilling activities in the locations selected for our study. It is also hereby confirmed that the field studies did not involve any endangered or protected species. Systematic fieldwork was carried out in the Achankovil, Pamba and Manimala river basins for the collection of data on various landform features and also for locating borehole sites for subsurface sample collection. Data retrieved from three boreholes and observations from two open wells have been used while addressing the peatland development in GPB (Fig 1). Selected field locations and sections from the Pamba river bank, Manimala River near Karippumuri kadavu and Achankovil river basin, near Vettiyar are illustrated (Fig 2).

The borehole core sediments were collected using rotary drilling unit fitted with Split Sediment Sampler—a method widely used for subsurface sample collection [10–13]. After documenting the gross lithological details, the borehole cores are sectioned at 10 cm intervals and sub-samples from selected depths were packed in neatly labeled Polyethylene bags for further laboratory analysis. Utmost care was taken in the field itself to avoid contamination during sub-sampling, packing and processing for various analytical procedures. The sediment samples were subjected to textural analysis [14]. The ternary diagram of Picard was used for classification of sediments [15]. The organic carbon (C_-org_) contents of the sediments were determined following the wet oxidation method of El Wakeel and Riley [16]. Average of triplicate measurements not differing 0.2% of the analyses were used in this study. Selected samples of the organic matter rich sediments were subjected to palynological examinations. The samples for recovering palynomorphs were processed by conventional method of separating organic walled microfossils from sediments [17–18]. Pollen identification of wet evergreen taxa is based on available database [19]. The retrieved sub-fossil log obtained from a nearby well was cut into transverse and longitudinal planes by using power driven automatic cutting machine having a diamond edge circular blade. Anatomical investigations of the specimen were carried out by Ground thin section method and Transfer techniques [20]. Photomicrographs were taken in a Canon Powershot digital camera. The nitrogen and carbon stable isotope composition (δ^15^N and δ^13^C_org_) of selected core samples was determined at Centre for Tropical Marine Ecology (ZMT), Bremen, Germany. The nitrogen isotope composition (δ^15^N) was determined with a Thermo Finnigan Delta Plus gas isotope ratio mass spectrometer after high temperature combustion in a Flash 1112 EA elemental analyzer. The carbon isotope composition (δ^13^C_org_) was determined similarly after removal of carbonates by adding 1N HCl and subsequent drying at 40°C. δ^15^N is given as ‰—deviation from the nitrogen isotope composition of atmospheric air and δ^13^C_org_ as ‰—deviation from the carbon isotope composition of the VPDB standard. The standard deviation of replicate measurements was 0.2‰ for both δ^13^C_org_ and δ^15^N. Radiocarbon dates used in present study were obtained from Birbal Sahni Institute of Palaeobotany, Lucknow, India. Sub fossil log specimen figured (MACS G 5419), and the micro-preparations in the form of figured slides (MACS G/ MF 2734 and MACS G/MF 2735) are housed in the repository of Geology and Palaeontology Group of Agharkar Research Institute, formerly known as Maharashtra Association for the Cultivation of Sciences (MACS), Pune, India.

## 4. Results and Discussion

### 4.1 Lithology and sediment characteristics

The core retrieved from Puvathur (9°20'25"N and 76°37'37"E) is 12.5 m long and is composed of four major lithounits, top clayey sand (3.0 m), followed subsequently by a mud dominated layer (3.0–6.8 m) which is intervened by a sand layer (0.9 m thick), peat with decayed wood (6.8–8.8 m), organic matter rich silty clay (8.8–11.0 m) and clayey sand (11.0–12.0). The entire sequence rests unconformably over the altered Archean crystalline basement (Fig 2). The sand, silt and clay contents in the top clayey sand are 57.05–65.92% (av. 61.40%), 15.20–18.06% (av. 16.98%) and 18.88–24.89% (av. 21.62%) respectively, while that of the mud dominated layer are 5.56–40.47% (23.02%), 22.66–46.29% (av. 34.48%) and 36.87–48.15% (av. 42.51%) respectively. The silty clay layer at the bottom accounts for 0.56–16.35% (av. 6.79%) of sand, 27.33–37.53% (av. 31.42%) of silt and 56.32–70.03% (61.79%) of clay (Table 2). It is followed by clayey sand with 50.14% sand, 17.48% silt and 32.38% clay.

**Fig 3:**
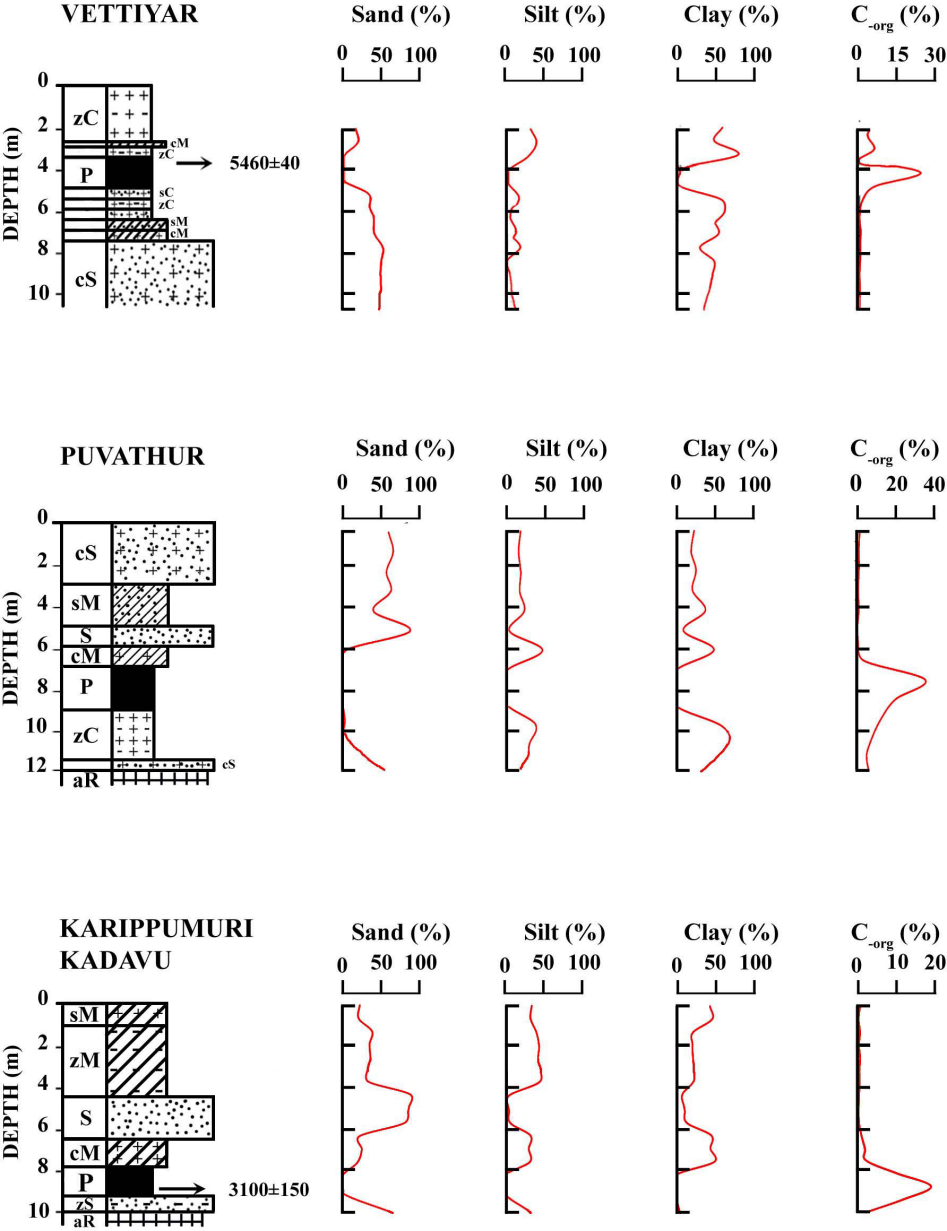
Downcore variation of sand, silt and clay contents of Vettiyar, Puvathur and Karippumuri kadavu borehole cores along with organic carbon (C_org_) content.

The Vettiyar borehole core (9°13'5" N and 76°36'15" E) is 10.5 m retrieved from the floodplains of Achankovil River. The core is composed of 7.4 m thick silt and clay dominated sediments followed by about 3.0 m thick sand dominated sediments (Fig 3). The top silt and clay dominant layer is interbedded at 3.4–4.9 m level by 1.5 m thick peat. The sand, silt and clay contents in the top silty clay and clayey mud layer are 1.89–20.74% (av. 13.54%), 24.40–35.63% (av. 29.36%) and 43.63–73.71% (av. 57.10%) respectively. The clay and mud dominated layer below the 1.5 m thick peat contains 34.23–44.25% (av. 38.15%) of sand, 2.57–13.35% (av. 7.76%) of silt and 52.42–56.69% (av. 54.10%) of clay, and 46.78–47.38% (av. 47.08%) of sand, 3.79–9.75% (av. 6.77%) of silt and 43.47–48.83% (av. 46.15%) of clay respectively. The sand, silt and clay contents in the bottom sand dominated layer are 54.63–57.90% (av. 56.19%), 0.52–16.96% (av. 7.34%) and 25.14–43.43% (av. 36.47%) respectively (Table 2). The peat bed at Vettiyar borehole core gives an age of 5460 ± 40 yrs BP at 3.5 m level.

The 10.5 m long bore hole at Karippumuri kadavu (9°23'48" N and 76°39'27" E) begins with 4.5 m thick, yellowish brown mud dominated sediments (1.0 m sandy mud and 3.5 m thick silty mud) which and is followed successively by 2.0 m thick sand, 1.0 m thick clayey mud and 1.2 m thick silty sand. The entire sequence rests over altered Achaean crystalline basement rocks (Fig 2). The sand, silt and clay contents in the mud dominated layers are 19.21–38.40% (av. 28.60%), 31.40–44.56% (av. 37.40%) and 20.11–48.37% (av. 34.00%), respectively. The intervening sand layer is composed of medium to coarse sand with 80.21–90.94% (86.33%) of sand, 1.83–7.12% (av. 4.17%) of silt and 6.90–12.67% (av. 9.77%) of clay (Table 2). The layer is exposed in the nearby river channel section (Fig 2). The organic rich clay/ peat underlying coarse to medium grade sand often embeds semi-carbonised log/ wood. The channel section at certain places shows large tree trunks that are oriented in the river flow direction. A few of such woods are radio carbon dated as 2881 ± 54 yrs BP, 2941 ± 102 yrs BP and 3389 ± 63 yrs BP. A wood sample embedded at the base of the carbonaceous clay/ peat has a radio carbon date of 3100 ± 150 yrs BP. All these point towards Middle—Late Holocene age for the peat bearing sediments in the GPB.

**Fig 4:**
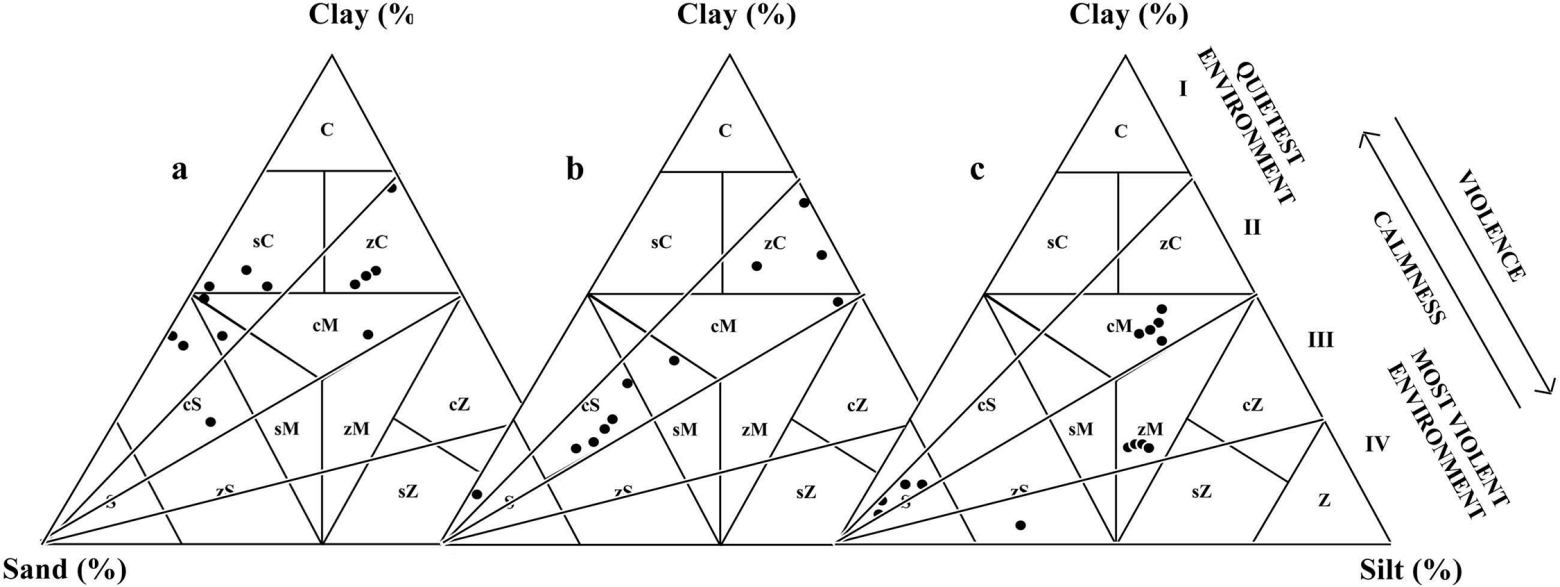
Sediment types based on Picard [15] of the borehole cores retrieved from the study area, a) Vettiyar, b) Puvathur and c) Karippumuri kadavu and also showing depositional environment suggested by Pejrup [21].

The ternary plots of sand, silt and clay in the hydrodynamic facies of Pejrup [21], for the cores of Puvathur and Vettiyar are illustrated (Fig 4A–4C). There are four different sectors in the ternary diagram indicating different energy fields from I to IV (quietest to most violent). In the present study, the ternary plots are segregated in the Sector I and Sector II indicating a comparatively low energy regime existed during the deposition of sediments in these locations. The Puvathur borehole core shows a coarsening upward sequence above the peat bed indicating a prograding type depositional environment with an increasing energy level during the sedimentation process. Whereas a reverse trend is noticed in the case of Vettiyar and Karippumuri kadavu borehole cores indicating sedimentation in a low energy regime, especially towards the upper part of the cores.

### 4.2 Analysis of peat and peat land vegetation

**Fig 5:**
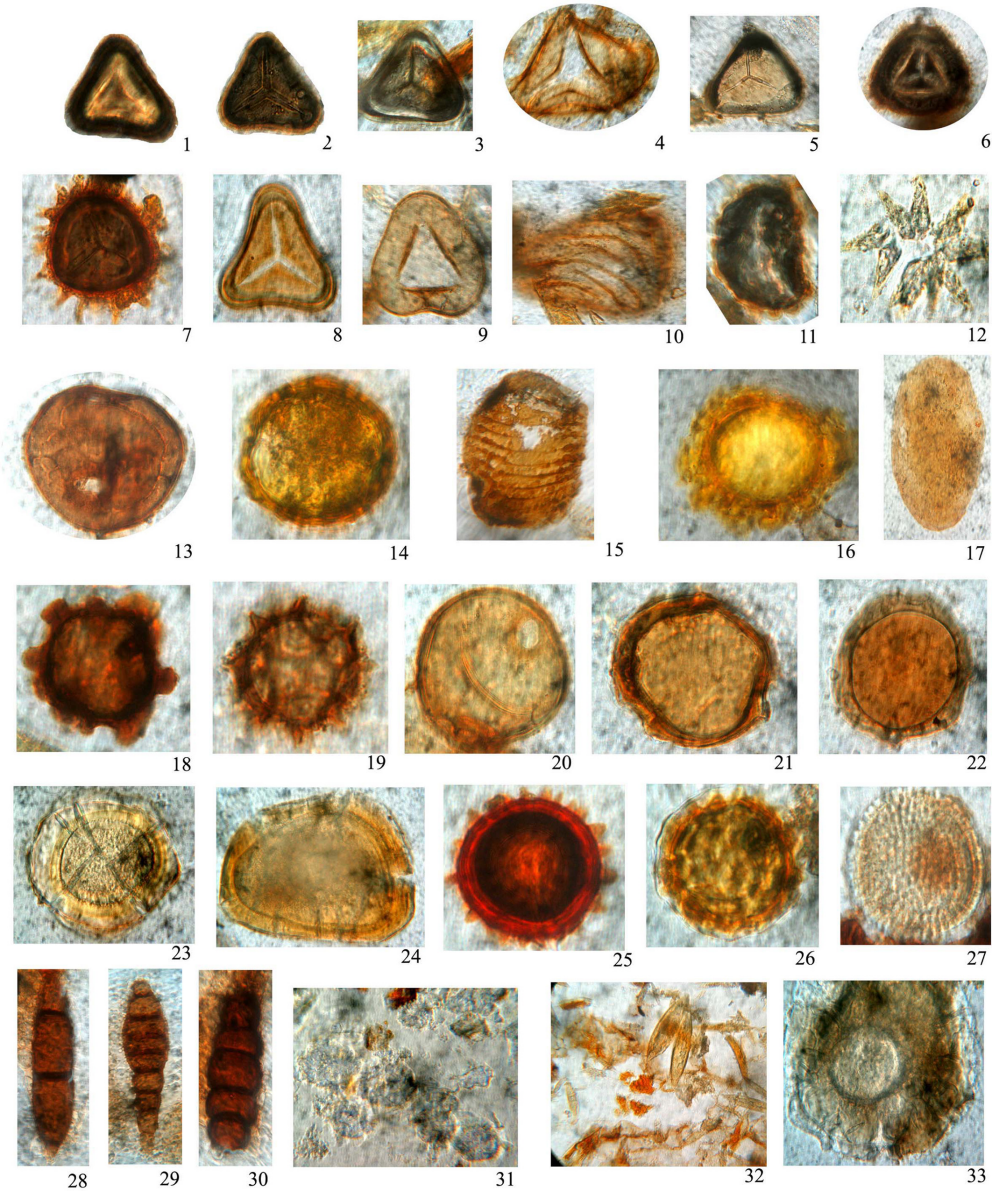
Palynological assemblage of Puvathur. (All photomicrographs magnified *ca* x 500 unless otherwise specified and ca x 1000 for fungal remains). 1, 2. *Pteridacidites* sp. (Pteridaceae). 3, 5. *Cyathidites* sp. (Cyatheaceae). 4, 6. *Ceratopteris thalictroides* (Parkeriaceae). 7. *Anemia phyllitides* (Anemiaceae). 8. *Cyathea* sp. (Cyatheaceae). 9. Trilete fern spore. 10. *Schizaeisporites multistriatus* (Schizaeceae). 11. *Polypodium vulgare* (Polypodiaceae). 12. *Pediastrum* sp. 13, 14, 22. Meliaceae pollen. 15. Acanthaceae pollen. 16, 25, 26. Malvaceae pollen. 17.? Acanthaceae pollen. 18. Euphorbiaceae pollen. 19. *Vernonia travancorica* (Asteraceae). 20, 21. *Cullenia exarillata* (Bombacaceae). 23, 24. Unidentified pollen. 27. *Croton* sp. (Euphorbiaceae). 28, 30. *Multicellaesporites* sp (Fungal spore). 29. *Pluricellaesporites* sp (Fungal spore). 31. *Botryococcus* sp. 32. Sponge spicules. 33. Thecamoeba.

Of the 12.50 m borehole about 2.0 m thick peat layer between 6.8 and 8.8 m is very conspicuous in Puvathur, about 1.50 m (3.4–5.9 m interval) thick at Vettiyar in Achankovil river basin and 1.50 m (8.0–9.5 m interval) at Karippumuri kadavu (Manimala River Basin). Higher concentration of organic carbon (C-org %) has been observed in the peat layer of the Puvathur core and it amounts to 26.15% at 6.90–7.00 m level. While in Vettiyar the peat layer at 3.9–4.0 m level is accounted for 23.9% of organic carbon (Fig 3; Table 2). The palynological assemblage essentially consists of pteridophytic spores, pollen of wet evergreen forests and fungal spores. However, the break in peat formation and change in facies has been indicated by freshwater plankton forms (*Botryococcus braunii*, *Staurastrum* spp., *Suriella* sp.). The palynoflorule of the peat layer is represented by local freshwater floodplain fern spores of *Ceratopteris thalictroides* (Parkeriaceae) and under storied pteridophytes (Pteridaceae, Cyathidaceae, Polypodiaceae etc.), freshwater swamp vegetation and also from the evergreen forest of highlands. Pollen of *Vernonia travancorica* (Asteraceae), *Cullenia exarillata* (Bombacaceae), *Calophyllum apetalum*, *Mesua ferrea* (Calophyllaceae), *Croton malabaricum*, *Mallotus* sp. (Euphorbiaceae), *Pterospermum diversifolium* (Malvaceae) represent the major plant constituents of the peat. The palynological observation and the facies changes recorded in Puvathur borehole have been presented in Table 3 and a few spores, pollen, plankton and fungal spores are illustrated (Fig 5). The palynological suite of peat layer indicates that peat developed in a fresh water swamp environment with high atmospheric humidity and the organic contribution has been essentially due to elements of evergreen forest vegetation. The occurrence of phytoplankton and *Pediastrum* sp. suggests freshwater stream activity within the peat swamps.

**Table 3:** Palynological observations in Vettiyar and Puvathur borehole cores.

Borehole Location	Sample Depth (cm)	Observations	Results
**Vettiyar**	220–230	Organic recovery is very high; palynological assemblage dominated by reworked pollen of *Ctenolophonidites* sp. The pollen shows high organic maturation and resembles to that of Warkalli Formation; charcoal particles in the form of charred cuticles of grasses frequent, indicating fire activity; a few pteridophytic spores are also observed.	Mainly continental / fresh water facies; evidence of erosional / reworking of Neogene sediments as the organic particles with high maturation level.
	490–500	Very good organic recovery and assemblage dominated by fungal spores; few pteridophytic spores too occur; reworked Neogene pollen *Ctenolophonidites* sp., occasionally seen.	Continental facies, mainly freshwater, sediments are of reworked type as the organic matter show heavy maturation; prevalence of high humidity due to fungal spores.
	960–970	Relatively poor organic recovery; non-pollen forms are seen; *Lakiapollis* sp. (resembling modern *Cullenia exarillata*) of Neogene observed.	Fresh water facies represented by plankton of *Staurastrum* sp., besides some desmids indicative of facies change and exposure of the sedimentary facies envisaged; erosion / reworking indicated due to Neogene pollen.
**Puvathur**	90–100	Assemblage is fair; organic contents include pteridophytic spores and pollen; phytoplankton mainly forms of *Staurastrum* spp., freshwater diatom- *Suriella* sp., pollen of Acanthaceae, Euphorbiaceae, Malvaceae, Meliaceae	Fresh water quieter environment where in phytoplankton community developed in a lake type system; pollen accumulation of *in situ* vegetation due to thick exine.
	240–250	Mainly pollen of higher and local vegetation and no plankton	Essentially a habitat of thick evergreen vegetation due to dominance of pollen having thick exine
	490–500	*Botryococcus braunii* is abundant; organic matter much less; a few Thecamoebians make up the entire assemblage; neither spore nor pollen observed.	Freshwater to brackish environment due to dominance of *Botryococcus* alga; however few thecamoebians indicate subsequent development of stress conditions. There is a change in ecological facies due to prevalence of fresh water aquatic environment.
	640–650	Abundance of desmids; few pteridophytic spores like *Pteridacidites* sp., *Ceratopteris thalictroides*, *Cyathidites* sp.; pollen of Euphorbiaceae, *Cullenia exarillata* Acanthaceae; even few phytoplankton, *Pediastrum* sp. make up the assemblage	Appears to be freshwater swamp environment where local vegetation is represented by pollen of evergreen plants and the occurrence of phytoplankton and *Pediastrum* suggests freshwater stream activity within the swamps. Higher atmospheric humidity envisaged due to pteridophyte spores
	690–700	Organic contents fair; pteridophytic spores and pollen with thick exine; fungal spores like *Meliolinites* sp. and other types common; cyanobacteria sp. observed due to enrichment of nutrients	Fresh water swamp environment with forest vegetation and prevalence of high humidity but not certainly lake as no plankton seen
	790–800	Fungal spores and pollen observed; Ascospores are also present; fungal spores indicative of high concentration of organic matter	Prevalence of forest vegetation and burial indicated with high humidity
	Paleosols from Wood cavity	Presence of Megascleres, fungal spores and Thecamoebians.	Accumulated scelerites indicating stress factor for a while when wood got exposed for a considerable period.

**Fig 6:**
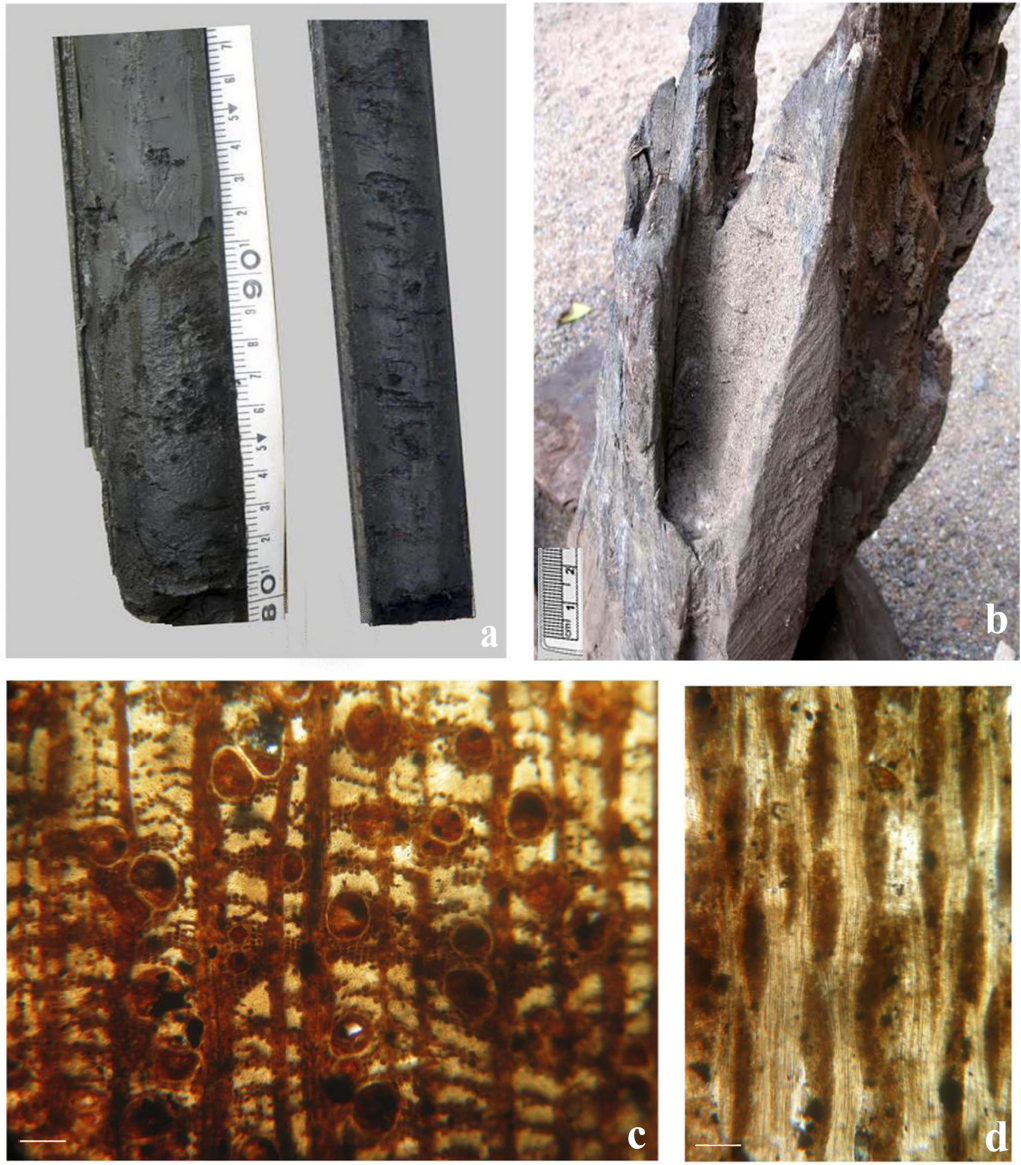
Subsurface sample and wood details from peatland in Pamba basin, Kerala. a. Split core having wood remains entombed in grey clay matrix obtained from a depth of 9.2 m below ground level at Puvathur. b. A piece of subfossil log retrieved from a well at Puvathur (MACS G 5419) c. Transverse section of *Cynometra* sp. Puvathur (PVTR) Scale bar = 250μm (MACS G/ MF 2734). d. Tangential longitudinal section of *Cynometra* sp. Puvathur (PVTR) Scale bar = 250μm (MACS G/ MF 2735).

The fossil log horizon observed is just below the peat layer (8.5–9.0 m) and the tree trunks here are too large and hard to be retrieved while coring. However, the tree trunk obtained from an adjacent open well belongs to Fabaceae and it has been provisionally identified as *Cynometra* sp. on the basis of wood anatomical characters (Fig 6C and 6D). It appears that these logs have been uprooted by sudden inundation of the habitat and preserved ‘*in situ’* without allowing much time for them to undergo decomposition and atmospheric oxidation. Such palaeofloods due to intensified monsoon must have accelerated the rate of sediment influx during the felling of the tree trunks and paved the way for formation of peat and subsequent peatland development towards Middle Holocene. However, the present ecology of these areas of buried tree trunks is unsuitable to support such large trees due to substantial modification of the forest and land cover into wetlands since Middle Holocene. Therefore, it can be concluded that these areas were covered by thick tropical rain forests which got destroyed during sediment build up in the area under high rainfall events. In fact these tree trunks can only be unearthed manually as they are being taken out while making open wells or canals for navigation and irrigation.

Analysis of organic content and sediments in Greater Pamba Basin has revealed that occurrence of peat and peatland development is attributed to the contributions of freshwater swamp and tropical evergreen forests that have been thriving in the plains of low elevations along the Kerala coast during the Holocene Climate Optimum (HCO). Such an environment and habitat must have been congenial for the formation of peat and peatland development which have been aided by intensified Monsoon [9]. The flooding of the forest probably took place as a result of intensified SW Monsoon coupled with sea level rise to the present level or slightly above, drastically increasing the sluggishness of the river flows leading to abrupt flooding of the forest habitat that supported the trees. The fallen trees were buried by the excessive sediment deposited by the rivers. Occurrence of such buried tree trunks and carbonaceous clay has been found to be very common in the coastal plains and in the low elevations all along the Kerala coast [3].

Considering the limited interval of peat to about 2.0–3.0 m or so and also taking into account its formation is about 1.0 mm per year the occurrence of peat and peatland development in the coastal plains, and river basins is rather very much restricted to a narrow time frame within the Middle Holocene (6.5–5.0 k yrs BP) when the environment and climate were very conducive for such development. The time frame coincides with intensified SW Monsoon which is usually referred to Holocene Climate Optimum. The same was observed in almost all the coastal deposits and river basins of Kerala studied earlier where in sudden burial of forest and organic matter were taken place due to flooding event towards Middle Holocene [3]. Anderson and Muller [22] had earlier reported tropical peat formation in Indonesia and that too falls within this narrow time frame. This kind of organic carbon due to peat, enriched the clay and soil, which in turn eventually imparted dark colour to sediments of the wetlands and surrounding landforms of Kerala. Such transformation of soil covers into sediments of enriched organic carbon eventually transformed the coastal plains into potential peatland to a considerable extent all along the Kerala coast. Accordingly the peatland development during the Middle Holocene has been found to be a unique formation and it is attributed to geological event associated with intensified SW monsoon.

### 4.3 Organic carbon, Nitrogen and Stable isotopes

Organic matter is an integral component of sediment and it reaches sedimentary environments from allochthonous and autochthonous sources. The contents of organic carbon, nitrogen and isotopic signatures in organic matter rich sediments are often used as an indicator of source sediments. The content of organic carbon varies from 0.1% to 26.2% with an average of 4.8% in the Puvathur core, <0.1% to 23.9% with an average of 4.3% in Vettiyar core and 0.3% to 19.0% with an average of 3.2% in the Karippumuri kadavu core.The content of nitrogen varies from 0.01% to 0.55% (av. 0.18%) in Puvathur and 0.04% to 0.72% (av. 0.31%) for Vettiyar core (Table 4). The C/N ratio also exhibits marked variation. The C/N ratio varies between 8.0 and 13.0 in the upper half of the Puvathur core, whereas it was two to three times higher in the lower half. The higher C/N ratio in the lower half points to a high input of terrestrial macrophytes, whereas, the lower ratio in the upper half indicates a higher organic matter contribution from aquatic algal sources [23–26]. This is further supported by the higher δ^13^C_org_ in the upper part of the Puvathur core (δ^13^C_org_ -24.5‰ to -23.1‰; Table 4). The δ^15^N also exhibits an almost similar pattern as that of δ^13^C_org_ with substantial higher values in the upper part (δ^15^N: 8.1‰ to 8.8‰) than that of the lower part (δ^15^N: 2.1‰ to 5.9‰). In combination with the very low organic carbon content this indicates a high degree of degradation [27–28]. However, not much significant trend as that of Puvathur core is noticed in the case of Vettiyar core. The layer at 5.4–5.5 m is highly degraded as compared to the upper layer (2.2–3.4 m level).

**Table 4:** Content of organic carbon (C_org_), nitrogen and C/N ratios in the sediments of Vettiyar and Puvathur along with the concentration of δ^15^ N and δ^13^ C_-org_.

Sample Depth (m)	Nitrogen (%)	C_org_ (%)	C/N	Δ^15^N (‰)	δ ^13^C_org_ (‰)
**Vettiyar**					
2.2–2.3	0.17	2.23	13.12	4.46	-25.26
3.3–3.4	0.72	11.75	16.32	2.09	-25.44
5.4–5.5	0.04	0.29	7.25	7.42	-26.76
**Puvathur**					
0.9–1.0	0.05	0.50	10.00	8.12	-23.09
2.9–3.0	0.03	0.24	8.00	8.82	-24.51
4.9–5.0	0.01	0.13	13.00	2.82	-28.02
6.9–7.0	0.55	26.15	47.55	2.13	-27.98
8.9–9.0	0.28	9.86	35.21	4.96	-27.81
11.4–11.5	0.14	2.87	20.50	5.94	-27.87

### 4.4 Consequences of Sea level and climate change in SW India

Sea level has played a significant role in the evolution of southwetern coast in general and the Greater Pamba Basin in particular bringing substantial modifications of the landscape and peatland development. Submergence of vast areas of forest cover and loss of sensitive ecosystem like freshwater *Myristica* swamps and mangrove vegetation are some of the features that have been identified along west coast of India since LGM [3, 29–31].The freshwater influx to watersheds and rivers largely depending on the monsoon system has been severely affected. Both Achankovil and Pamba rivers have been subjected to hydrodynamic regimes since past several thousand years as a result of monsoon variability [3]. During the LGM (20–18 k yrs BP) event the Arabian Sea experienced a lowering of its level to the tune of 100–120 m. The consequences of this event had left the extensive coastal stretch and adjoining inland forests to a considerable extent exposing them to monsoon vicissitudes. The excessive rainfall could have caused some inundation of the low-lying lands. The rivers due to increased gradient become much more erosive in nature causing widespread removal of sediments already deposited except from those in inland lakes and depressions. As a consequence, the rivers too have lost their original vigour and changed course towards Vembanad Lake as seen in the satellite imageries [32]. The change of its course has left vast flood plains, a few islands and small water bodies before it finally reaches to Kuttanad. Anthropogenic factor, especially sand mining activities added new dimension to the evolution and current status of Pamba towards Late Holocene.

### 4.5 Peat as potential Palaeoclimate archive

Peat layers, woody peat/carbonized logs and sub-fossil log horizons observed at different locations in the wetlands and floodplains of GPB indicate that the formation of peat and peatland development is essentially restricted to freshwater swamps during the early part of Middle Holocene unlike the tropical Southeast Asian peatlands that occur along maritime fringes, deltaic areas and at slightly higher elevations of inland since Late Pleistocene [33].The virtual transformation of the forests into peat and buried palaeoforests that remain submerged within the wetlands and in the lowlands indicate that peat formation and uniform peatland development took place in a narrow time frame when the rising sea levels stabilized and fell slightly during the Middle Holocene (6.5–5.0 k yrs BP). A combination of factors like the low topographic relief, impermeable substrates and high rainfall have provided favourable conditions for accumulation of organic material and subsequent conversion into thick deposits of peat. Evidence for such an intensified monsoon and higher rainfall leading to virtual displacement of forest vegetation and converting into palaeoforest in SW India has already been dealt by Kumaran et al. [3] and an increased Indian Summer Monsoon (ISM) and subsequent vegetation development of mixed C3/C4-plants during the Early to Middle Holocene from the Bengal region [34]. The relative stability of sea level as well as the intensification of monsoon strength led to increasing rainfall towards Middle Holocene which in turn favoured the formation of freshwater swamps and subsequent accumulation of peat in the lowlands. The palynological assemblage, carbon isotope signatures and the sub-fossil log evidence suggest that peat has been derived from the tropical evergreen forests when the vegetation was subjected to severe floods and subsequent burial. This uniform development of peat in almost all the lowlands and floodplains in Kerala has transformed a major part of Kerala coast into a perpetual peatland towards Early Middle Holocene. The peatland development has converted a major part of the lowlands into one of the best known carbon sinks in this part of south-western India. Evidence of peat from different locations in Kerala and the time frame of peat development provided in the Table 5 indicate that peat formation is restricted to narrow time frame when the environmental conditions were conducive for such events.

**Table 5:** Radiocarbon dates from the coastal sedimentary environments of Kerala with reference to peat.

Sr. No.	Location	Depth (m)	Material	^14^C date in yrs BP	Reference
1.	Thalasseri, SSE of Kannur	2.00	Peat, BS 711	7,230 ± 120	[4]
2.	Thannisseri, Irinjalakuda	2.00	Peat, BS 689	6,420 ± 120	[4]
3.	Poovathumkadavu,15 km NNW of Kochi	2.00	Peat	3,390 ± 110	[41]
4.	Poovathumkadavu, 15 km NNW of Kochi	3.00	Peat	5,520 ± 160	[41]
5.	Poovathumkadavu15 km NNW of Kochi	6.50	Peat	6,720 ± 70	[41]
6.	Poovathumkadavu15 km NNW of Kochi	24.00	Peat	7,450 ± 120	[41]
7.	Valoor	2.00	Peat	3,390 ± 110	[41]
8.	Valoor	3.00	Peat	5,520 ± 160	[41]
9.	Willigton Island	16.75	Peat	8,315 ± 125	[42]
10.	Changa (Aruvikkara)	2.50	Peat	3300 ± 90	[43]
11.	Annallur	4.0	Peat	6630 ± 120	[44]
12.	Iranimangalam, 12 km ESE of Aleppey	-	Peat	7820 ± 120	[45]
13.	Vettiyar	4.0	Peat	5460 ± 40	Present study
14.	Karippumurikadavu	9.0	Peat	3100 ± 150	Present study

Peat deposits and fluvial sediments are very good terrestrial archives for palaeoclimate appraisal and also for ascertaining the monsoon variability records of Late Quaternary period [35]. Compared to marine and lacustrine sedimentary records, terrestrial archives are seldom tested for high resolution monsoon records and for palaeoclimate reconstruction since Last Glacial Maximum (LGM) due to paucity of Late Quaternary deposits in Peninsular India. Except the Nilgiri peat bogs from southern India [36], a few locations in South Kerala Sedimentary Basin (SKSB) [5, 37], and in Konkan [30] terrestrial archives that cover the time span of Late Pleistocene–Holocene have been rarely retrieved for palaeoclimate appraisal and as such there exists a wide gap in our knowledge of monsoon dynamics and its impact on landforms and vegetation since LGM. However, there have been attempts in the last decade to decode the terrestrial archives of SW India using pollen and plant macrofossil assemblages [3, 38] and recently from the Bengal region [34]. There is no doubt that both Southwest (SW) and Northeast (NE) monsoons have brought in significant impact on the hydrological regimes and their consequences upon the landscape and vegetation dynamics ever since uplift of the Himalayas and other mountain building processes began 8 million years ago [39]. Considering the lack of knowledge on Late Pleistocene and Early Holocene from SW India the present communication based on signatures decoded from the terrestrial archives holds immense potential for addressing the geological events and consequent climate impact on landforms and vegetation.

## 5. Summary

The finding of subsurface peat and sub-fossil logs in Greater Pamba Basin implies that the floodplains and sub-coastal areas (5–10 m asl) in SW India had adequately provided essential topographic relief, impermeable substrates and environmental conditions favorable for accumulation of large volume of organic materials during the Middle Holocene when the rising sea levels stabilized and fell slightly (6.5–5.0 k yrs BP). Such an environmental set up eventually led to development of one of the relatively youngest tropical peatlands in the extreme southern tip of India and has operated as long term carbon sink. The accrued data indicate that the peat is ombrotrophic and is essentially derived from the freshwater swamps and evergreen forests. The high rainfall and massive floods coupled with a rising sea level must have inundated > 75% of the coastal plain land converting it into a veritable lagoon—lake system that eventually led to abrupt termination of the forest ecosystem and also converted the floodplains into peatland where accumulation of peat almost to 2.0–3.0 m thickness in coastal lowlands and river basins during the shorter interval in the Middle Holocene. The virtual transformation of the forests into peat and buried palaeoforests that remained submerged within the wetlands and in the lowlands indicates that peat formation and uniform peatland development took place in a narrow time frame towards Middle and Late Holocene unlike the uninterrupted formations throughout the Holocene in the Southeast Asian region. Considering the tropical coastal lowlands and associated peatlands are excellent repositories of carbon, they are very important for regional carbon cycling and habitat diversity. The alarming rate of land modification and development is destabilizing these carbon pools resulting in large scale carbon emissions to the atmosphere and loss of low-latitude peat palaeorecords. Since the peat bed and peatland hold immense potential in carbon sequestration and climate change study these palaeoresources are to be focused further. Therefore, palaeoenvironmental studies of peatlands of south-western India need to be investigated.

## 6. Conclusions

Tropical Peatland development in the Southern Peninsular Indian region during the Holocene has been attempted for the first time based on subsurface sediments obtained from the sub-coastal lands and floodplains of Kerala in SW India. The signatures of peat and peatland development have been decoded with the help of multiple proxies. Unlike the older records, the Holocene peat has been essentially formed in a freshwater swamp environment and the peatland development has started in the incurves of meander loops of fluvial channels since the beginning of Middle Holocene subsequent to the sea level rise around 7,000–6,500 yrs B.P. The sea level rise together with the excessive rainfall during this period could have provided conducive geo-environmental set up for river meandering in the coastal lands and adjoining parts of the midlands which in turn favoured peatland development in its incurves. The interruption of peatland development observed in SW India during the Holocene is attributed to the combined effects of climate variability and sea level oscillations.

## References

[1] Rajagopalan G, Sukumar R, Ramesh R, Pant RK, Rajagopalan G (1997) Late Quaternary vegetational and climate changes from tropical peats in southern India—extended record upto 40,000 years BP. Current Science 73: 60–63.

[2] Phadtare NR, Pant RK (2006) A century-scale pollen record of vegetation and climate history during the past 3500 years in the Pinder Valley, Kumaon Higher Himalaya, India. Journal Geological Society of India 68: 495–506.

[3] Kumaran KPN, Padmalal D, Nair KM, Limaye RB, Guleria JS, Srivastava R, et al (2014) Vegetation response and landscape dynamics of Indian Summer Monsoon variations during Holocene: An eco-geomorphological appraisal of tropical evergreen forest subfossil logs. PLOS ONE doi: 10.1371/journal.pone.0093596 10.1371/journal.pone.0093596 PMC3984104 24727672

[4] Rajendran CP, Rajagopalan G, Narayanaswamy (1989) Quaternary geology of Kerala: Evidence from radiocarbon dates. Journal Geological Society of India 33, 218–222.

[5] Kumaran KPN, Nair KM, Shindikar M, Limaye RB, Padmalal D (2005) Stratigraphical and palynological appraisal of Late Quaternary mangrove deposits of the west coast of India. Quaternary Research 64 (3): 418–431.

[6] Narayana AC (2007) Peat deposits of west coast of India: implications for environmental and climate changes during Late Quaternary. Journal of Coastal Research 50: 683–687.

[7] Nair KM, Padmalal D (2003) Quaternary Sea level oscillations, Geological and Geomorphological evolution of South Kerala Sedimentary Basin. PCR ESS/23/VES/6/98; Report submitted to DST, GOI., pp. 1–60.

[8] Nair KM, Padmalal D, Kumaran KPN (2006) Quaternary Geology of South Kerala Sedimentary Basin—An Outline, Journal of Geological Society of India 67: 165–169.

[9] Nair KM, Kumaran KPN, Padmalal D (2009) Tectonic and Hydrologic Control on Late Pleistocene Holocene Landforms Palaeoforest and Non-Forest Vegetation, Southern Kerala. Project Completion Report, Kerala State Council for Science Technology and Environment, Thiruvananthapuram, pp. 1–83.

[10] Limaye RB, Kumaran KPN, Nair KM, Padmalal D (2010) Cyanobacteria as potential biomarker of hydrological changes in the Late Quaternary sediments of South Kerala Sedimentary Basin, India. Quaternary International 213: 79–90.

[11] Nair KM, Padmalal D, Kumaran KPN, Sreeja R, Limaye RB, Srinivas R. (2010) Late Quaternary evolution of Ashtamudi, Sasthamkotta lake systems of Kerala, south west India. Journal of Asian Earth Sciences 37: 361–372.

[12] Padmalal D, Kumaran KPN, Nair KM, Baijulal B, Limaye RB, Srinivas RR, et al (2011) Evolution of the coastal wetland systems of SW India during Holocene: Evidence from marine and terrestrial archives of Kollam coast, Kerala. Quaternary International 237: 123–139.

[13] Padmalal D, Nair KM, Kumaran KPN, Sajan K, Vishnu Mohan S, Maya K, et al (2013) Climate and Sea level changes in a Holocene Bay Head Delta, Kerala, and Southwest coast of India In: Sundaresan J. Sreekesh S, Ramanathan AL, Sonnenschein L, Boojh R, Climate Change and Island and coastal vulnerability, Springer, Dordrecht, The Netherlands and Capital Publishing Company, New Delhi, India, pp. 191–208, doi: 10.1007/978-94-007-6016-5_13

[14] Lewis DW (1984) Practical sedimentology Hutchinson Ross Publishing Company, Pensylvania.

[15] Picard MD (1971) Classification of fine grained sedimentary rocks. Journal of Sedimentology and Petrology 41: 179–195.

[16] El Wakeel SK, Riley JP (1957) The determination of organic carbon in marine muds. J. Du Conseil International Pour l’exploration De la Mer 22: 180–183.

[17] Moore PD, Webb JA, Collinson ME (1991) Pollen analysis Blackwell Scientific Publication, Oxford.

[18] Traverse A (2007) Palaeopalynology, second ed. Springer, Dordrecht, The Netherlands.

[19] Tissot C, Chikhi H, Nayar TS (1994) Pollen of wet evergreen forests of the Western Ghats, India. Institut Francais de Pondichery, pp. 1–133.

[20] Hass H, Rowe NP (1999) Thin sections and wafering In: Jones TP, Rowe NP, Fossil plants and spores: modern techniques, Geological Society, London, pp. 76–81.

[21] Pejrup M (1988) The triangular diagram used for classification of estuarine sediments: a new approach In: de Boer P.L. (Ed.), Tide Influenced Sedimentary Environment and Facies. Riedal Publishing, pp. 289–300.

[22] Anderson JAR, Muller J (1975) Palynological study of a Holocene peat and a Miocene coal deposit from NW Borneo. Review of Palaeobotany and Palynology 19: 291–351.

[23] Hedges JI, Clark WA, Quay PD, Richey JE, Devol AH, Santos Umberto de M (1986) Compositions and fluxes of particulate organic material in the Amazon River. Limnology and Oceanography 31: 717–738.

[24] Meyers PA (1994) Preservation of elemental and isotopic source identification of sedimentary organic matter. Chemical Geology 114: 289–302.

[25] Mahapatra DM, Chanakya HN, Ramachandra TV (2011) C:N ratio of Sediments in a sewage fed Urban Lake. International Journal of Geology 5(3): 86–92.

[26] Vijayaraj R, Achyuthan H (2015) Distribution of Sediments and Organic Matter Source: Berijam Lake, Tamil Nadu. Journal Geological Society of India 86: 620–626.

[27] Jennerjahn TC, Ittekkot V, Arz HW, Behling H, Pätzold J, Wefer G (2004a) Asynchronous terrestrial and marine signals of climate change during Henrich events. Science 306: 2236–2239. 15576572 10.1126/science.1102490

[28] Jennerjahn TC, Ittekkot V, Klöpper S, Adi S, Nugroho SP, Sudiana N et al (2004b) Biogeochemistry of a tropical river affected by human activities in its catchment: Brantas River estuary and coastal waters of Madura Strait, Java, Indonesia. Estuarine Coastal and Shelf Science 60: 503–514.

[29] Limaye RB, Kumaran KPN, Padmalal D (2014) Mangrove habitat dynamics in response to Holocene Sea level and climate changes along southwest coast of India. Quaternary International 325: 116–125.

[30] Kumaran KPN, Limaye RB, Punekar SA, Rajaguru SN, Joshi SV, Karlekar SN (2013) Vegetation response to South Asian Monsoon variations in Konkan, western India during the Late Quaternary: Evidence from fluvio-lacustrine archives. Quaternary International 286: 3–18.

[31] Srivastava G, Trivedi A, Mehrotra RC, Paudayal KN, Limaye RB, Kumaran KPN et al (2016) Monsoon variability over Peninsular India during Late Pleistocene: Signatures of vegetation shift recorded in terrestrial archive from the corridors of Western Ghats. Palaeogeography, Palaeoclimatology, Palaeoecology 443: 57–65.

[32] Padmalal D, Kumaran KPN, Limaye RB, Baburaj B, Maya K, Vishnu Mohan S (2014) Effect of Holocene climate and sea level changes on landform evolution and human habitation: Central Kerala, India. Quaternary International 325: 162–178.

[33] Page S, Wust R, Banks C (2010) Past and present carbon accumulation and loss in Southeast Asian peatlands. Pages News 18 (1): 25–27.

[34] Contreras—Rosales LA, Jennerjahn T, Tharammal T, Meyer V, Lückge A, Paul A, et al (2014) Evolution of the Indian Summer Monsoon and terrestrial vegetation in the Bengal region during the past 18 ka. Quaternary Science Reviews 102: 133–148.

[35] Singhvi AK, Kale VS (2009) Palaeoclimate studies in India: Last ice age to the present Indian National Science Academy, New Delhi, pp. 1–34.

[36] Sukumar R, Ramesh R, Pant RK, Rajagopalan G (1993) A δ^13^C record of Late Quaternary climate change from tropical peats in southern India. Nature 364: 703–706.

[37] Kumaran KPN, Limaye RB, Nair KM, Padmalal D (2008) Palaeoecological and Palaeoclimate potential of subsurface palynological data from the Late Quaternary sediments of South Kerala Sedimentary Basin, southwest India, Current Science 95: 515–526.

[38] Ajaykumar B, Sreedharan K, Mahesh Mo, Paul J, Thomas AP, Nair PKK (2012) Evaluation of the Holocene environmental changes of the southwest coast, India: A palaeopalynological approach. Journal of Earth System Science 121(4): 1093–1103.

[39] Valdiya KS (2002) Emergence and evolution of Himalayas: Reconstructing history in light of recent studies. Progress in Physical Geography 26(3): 360–399.

[40] Milliman JD, Syvitski JPM (1992) Geomorphic / tectonic control of sediment discharge to the ocean: The importance of small mountainous rivers. Journal of Geology 100: 325–344.

[41] Shajan KP (1998) Studies on Late Quaternary sediment sand sea level changes of the central Kerala coast, India. Ph.D. thesis (Unpublished), Department of Marine Geology and Geophysics school of Marine Sciences, Cochin University of Science and Technology Cochin, 155 p.

[42] Agrawal DP, Gupta SK, Kusumgar S (1970) Radiocarbon dates of Quaternary Samples. Current Science 30: 219–222.

[43] Tomalin V, Selvakumar V, Nair MV, Gopi, PK (2004) The Thaikkal-Kadakkarapally boat: an Archeological example of Medieval shipbuilding in the western Indian Ocean. International Journal of Nautical Archaeology 33: 253–263.

[44] Santosh V (2006) Environmental impact assessment of Tile and Brick Clay mining from Chalakudy and Periyar River Basins, Kerala, India.Ph.D. thesis, Kerala University, India.

[45] Pawar SD, Venkataramana Thomas Mathai, Mallikarjuna C, (1983) Systematic geological mapping around Shertall, Vikkom, Alleppey, Kottayam and Pandalam areas in parts of Alleppy and Kottayam Districts: Unpublished Report of the Geological Survey of India.

